# Health Care Apps Reported in Newspapers: Content Analysis

**DOI:** 10.2196/10237

**Published:** 2018-10-22

**Authors:** Abdel Qader Al Bawab, Fahad AlQahtani, James McElnay

**Affiliations:** 1 Faculty of Pharmacy Al-Zaytoonah University of Jordan Amman Jordan; 2 Clinical and Practice Research Group School of Pharmacy Queen's University Belfast Belfast, Northern Ireland United Kingdom

**Keywords:** apps, newspaper articles, newspapers, health

## Abstract

**Background:**

Newspapers are considered one of the most viewed and influential media sources in both the United Kingdom and United States. However, information about how newspapers portray health care apps to the readers has been lacking.

**Objective:**

This study investigated the reporting on health care apps in newspapers published in the United Kingdom and United States.

**Methods:**

The Nexis UK database was used to identify and select relevant articles. Systematic content analysis of the articles that met the inclusion criteria (articles of any format that contained reference to health care apps or medical apps) within the highest circulated newspapers in the United Kingdom and United States over a period of 10 years (2006-2015) was conducted. Interrater reliability of coding was established using a 10% sample of the chosen articles.

**Results:**

A total of 220 (151 UK and 69 US) relevant newspaper articles were retrieved. Health care apps were most frequently reported on in the Daily Mail and The Guardian (UK newspapers) and in the New York Times and the Washington Post (US newspapers). An exponential rise in published scientific articles (PubMed) on health care-related apps was noted during the study period. A total of 26.4% (58/220) and 19.1% (42/220) of the retrieved newspaper articles appeared in the features and main news sections, respectively. General information about health care apps was the main theme coved by the newspapers (45.9%, 101/220). Most of the articles represented a societal point of view (72.3%, 159/220). The main focus of the articles was on general health matters (48.2%, 106/220) and specific disease matters (36.8%, 81/220). Diabetes was the most frequently mentioned disease in the articles. A high proportion (91.4%, 201/220) of the articles mentioned benefits of using health care apps mainly for personalized care, whereas 24.1% (53/220) of the articles commented on related risks such as anxiety and confidentiality issues. Almost half (45.9%, 101/220) of the articles mentioned potential facilitators to the use of apps; less than 10% (16/220) discussed barriers. Most of the articles (83.6%, 184/220) were judged as having balanced judgment on the present topic and more than half (60.0%, 132/220) of the articles were judged to be of generally low quality.

**Conclusions:**

Health care apps were not widely reported in newspaper articles in the United Kingdom and United States over the study period; however, there appeared to be much more recent interest. Characteristically, the articles focused more frequently on societal impact and on general health rather than on disease-specific apps.

## Introduction

Health care apps are defined by the US Food and Drug Administration (FDA) as “software programs that run on smartphones and other mobile communication devices in order to, for example, provide health information to the public, provide other health-related support to patients and health care professionals and remotely transfer data to health care providers.” These apps can also be used to facilitate decision making and provide the opportunity for consultations to take place remotely [1].

Mobile phone apps are becoming increasingly popular globally and are being used by a substantial proportion of the population both in the developed and the developing world. They are used on a daily basis to communicate with friends and relatives, perform online shopping, read news, and for monitoring health-related parameters including diet, exercise, and sleep. Such apps can have a positive impact on promoting improved health and well-being and are increasingly being embraced by health care professionals [2,3].

The two major app stores are the iPhone Apple Store, which was launched in July 2008, and Google Play, which was introduced to the public in April 2010 [4]. Apps for health care-related use were initially introduced in 2011 within the Apple Store, which led to their widespread uptake across both healthy and ill individuals [5]; by the beginning of 2014, around 1 million general apps were available [6]. Health apps are used on mobile phones to track, for example, an individual’s heart rate, exercise, weight, food consumption, and sleep patterns. Apps have also been used in many research studies to collect data, send patient reminders, and to convey educational or motivational messages [7]. Health care apps are also becoming increasingly used in health care management to provide individual and population levels of support to health care recipients [8]. Therefore, mobile devices and information technology have been combined to promote health care and reinforce disease management. The National Health Service (NHS) health apps library was introduced in 2013 to provide information on recommended medical apps to help members of the public and patients in the selection of apps to manage their health. App developers can send their apps for review by the NHS library. Apps that are deemed beneficial (eg, the iBreastCheck app) are then added to the library list [9]. As detailed in a recent report, the four most frequently downloaded apps from the NHS library were related to general health and well-being, mainly related to the topics of fitness, drink trackers, healthy food, and quitting smoking. The fifth most downloaded app was for recording and tracking blood pressure [10]. One survey in the United States showed that approximately 25% of adult Americans use one or more mobile phone apps to monitor their health and 33% of health care professionals have recommended at least one app to their patients [11].

As well as widespread use by the public, health care providers increasingly use mobile phones to access health information at the point of patient care, such as electronic clinical decision-making programs, laboratory systems, and medical resource tools (eg, the Medscape app) [1,9]. According to a UK survey published in 2012, 74.8% of junior doctors and 79% of medical students owned a mobile phone, with the majority of both groups reporting that they used between one and five health care apps on their mobile phones [4]. All these numbers have likely increased in the interim within a burgeoning information technology marketplace. Many health apps are currently used for supporting disease management and monitoring patient health care, such as the Mobile MIM app that has been approved by the FDA as a portable x-ray scan viewer. With the high-definition screens available on the new generation of mobile phones, it has been reported that the assessment of images on mobile phones can be as effective as their evaluation at a workstation [12].

Health care apps can also facilitate access to a patient medication regimen and can be used to arrange an appointment with a physician. Apps can be utilized to educate patients and help change their behavior; for instance, healthy food apps can be used to monitor an individual’s behavior related to calorie intake and provide advice about healthy eating habits including fat and carbohydrate content of different food items [3,13]. Recent advances in mobile technologies have opened up new approaches to support health care delivery and patient education. These approaches have the potential to encourage patients to be more actively involved in their own health care and to be part of the decision-making process [8]. Some health apps are focused only on specific diseases such as the ophthalmology (“Eye Handbook” app [14]) and “Diabetes: M” for diabetes [15].

Most patients do not visit their general medical practitioner regularly; therefore, the public in general often gain information on health care innovations via the mass media (eg, television, radio, newspapers, magazines). The internet is also frequently searched to obtain answers to specific health queries. Newspapers are one of the main sources for providing health knowledge and medical information passively to the public [16,17]. In addition, it has been reported that although different mass media outlets differ in the quantity of coverage, newspapers are equivalent to other forms of news media in terms of content [18,19]. The print media is widely accessible to most people and is available at low cost [20]. Although newspapers are traditionally available in hardcopy, increasing numbers of the public access newspapers online. Research has revealed that mass media has a positive impact on changing behavior; for example, relating to alcohol use, diet, smoking, and breast feeding [21,22]. Furthermore, the impact of the media on cancer awareness has been well documented. Several studies reported increased screening for cancer and improved awareness in the United States and Australia after mass media campaigns involving newspapers [23,24].

Print media is a key source of health information that influences public understanding of health care-related matters. Print media can also influence public opinion and perceptions regarding a particular topic [25,26], and is an important vehicle by which health-related information is diffused within society [27]. Interestingly, public health professionals have a bimodal relationship with the media; they use health media to influence health practice, while at the same time they have to counteract the influence when the media promotes unhealthy or nonevidence-based practices [28]. Therefore, print media significantly contributes to the definition and understanding of health-related matters and health care practice. It has also been reported that policy responses to health-related issues are affected by media coverage of the problem rather than the true impact of the problem, and both policymakers’ perceptions and the public’s acceptance of possible policy responses are substantially influenced by the media [29]. The influence of the media on health-related issues has been reported frequently in the literature regarding, for example, obesity [30], smoking cessation [27], immunization in children [31], and AIDS [32].

The media of course can present material to the public within a particular framework and with a particular emphasis or slant, and indeed there is extensive literature on this effect (framing theory). Through the use of differing “framing” approaches, journalists can selectively influence how a particular piece of information is interpreted by the reader [33-35].

With the knowledge that newspapers represent an important source of health-related information [36], and because no published research has investigated newspaper reporting on health care apps, the aim of this study was to explore what the general public have been told about health care apps within published newspaper articles in the United States and United Kingdom over a 10-year period (2006-2015) and to analyze the content of the articles.

## Methods

### Study Design

**Figure 1:**
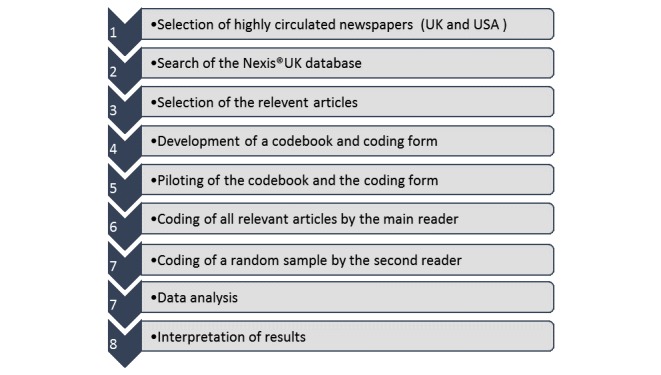
Flow diagram of content analysis methodology used in this study. UK: United Kingdom, US: United States.

We conducted a systematic content analysis of articles contained in the highest circulated newspapers in the United Kingdom and United States that dealt with health care apps (2006-2015). A summary of the methodology is presented in Figure 1.

#### Newspaper Selection

The electronic archive of published international newspapers (Nexis UK database) was used as the source of the selected articles. A purposive sample of the 10 daily UK newspapers (including their Sunday equivalents) with the highest circulation according to the Audit Bureau of Circulation Ltd [37] at the commencement date of the study (June 2015) was selected. This sample comprised *The Sun* (*The Sun on Sunday*), *Daily Mail* (*Mail on Sunday*), *Daily Mirror* (*The Sunday Mirror*), *Daily Star* (*Daily Sunday Star*), *The Daily Telegraph* (*The Sunday Telegraph*), *The Daily Express* (*The Sunday Express*), *Daily Record* (*Daily Sunday Record*), *The Times* (*The Sunday Times*), the *i*, and *The Guardian* (*The Observer*).

In an equivalent manner, a sample of the top 10 daily US newspapers, ranked by the total average circulation [37], was identified which consisted of *USA Today*, *The New York Times*, *Los Angeles Times*, *The Washington Post*, *New York Post*, *New York Daily News*, *Orange County Register*, *Newsday*, *The Denver Post*, and *Tampa Bay Times*. All except *USA Today* (published on weekdays) are published daily and on Sundays. Several highly circulated US newspapers, including the *Dallas Morning News*, *Chicago Tribune*, and *Chicago Sun-Times*, were not included in the study because they were not available in the Nexis database at the time the research was being carried out.

#### Search Strategy, Inclusion, and Exclusion Criteria

A systematic approach using different search terms to maximize relevant article retrieval was used. The search terms that yielded the highest number of articles from the Nexis UK database were: (health apps OR mobile medical apps OR smartphone health apps OR fitness apps OR exercise apps OR health care apps OR diet apps OR weight loss apps OR blood pressure apps OR diabetes apps). The addition of “OR asthma apps,” for example, did not increase the number of retrieved articles.

Articles of any format, such as news articles, editorials, and letters to the editor, were included in the analysis if they contained reference to health care apps or medical apps. Articles were excluded from the analysis if the health care apps were only mentioned briefly (ie, less than 10% of the article content), if the article focused only on health care app announcements (eg, advertising fitness apps), or in the case of duplication (eg, in a daily and Sunday equivalent), in which case only the article with the highest word count was included.

#### Data Extraction and Coding Frame

Based on previous work utilizing systematic content analysis, an a priori standardized coding book was developed. A pilot exercise was conducted in which 10 articles, chosen randomly, were coded independently by two of the authors (FA and JM) and the results were compared. During this pilot, minor modifications were made to the coding framework to increase code specificity. The final coding framework contained the following three main sections:

1. Basic information: the name of the newspaper, the title of the article, the year of publication, and the positioning of the article within the newspaper.
2. Article content: the themes covered, the perspective from which the article was written, the focus of the article, and benefits and risks or facilitators and barriers relating to the use of the health care apps outlined in the article.
3. Judgment and rating: the subjective judgment and rating of the reviewer on the main emphasis of the article, claims, and quality of information.

One researcher (FA) retrieved and read all the relevant archived newspaper articles and used the final coding form to manually code each article. To obtain a quantitative estimate of the coding reliability, a 10% random sample (random.org) of articles (n=22) were coded by the second reviewer (JM). Cohen kappa scores were calculated to evaluate interrater agreement for codes with mutually exclusive answers. The PubMed database was also searched for relevant articles over the same study period to gain an insight on how trends in newspaper reporting followed trends in scientific research publications on mobile apps used within health care. Following data extraction, codes were entered into SPSS version 21 for the analysis of trends and for comparing the variables between countries (United Kingdom and United States). Descriptive statistics were used to summarize the data. The Mann-Whitney *U* test was used to test for differences between means of continuous variables. Differences in the reporting of categorical variables were assessed using the chi-square test (χ^2^) or the Fisher exact test, as appropriate. Statistical significance was set at *P* ≤.05.

## Results

### Article Frequency

**Figure 2:**
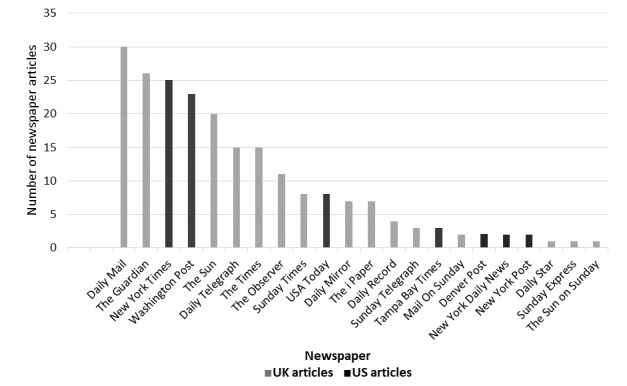
Frequency of articles about health-related apps in UK and US newspapers from 2005 to 2016.

The initial search yielded a total of 714 UK and US newspaper articles reporting on health care apps between 2006 and 2015. After removing duplicate articles, 689 articles were retrieved, of which 220 articles met the inclusion criteria; 151 articles were published in UK newspapers and 69 articles were published in US newspapers. The distribution of the articles across the different newspapers is presented in Figure 2. In the United Kingdom, the *Daily Mail* and *The Guardian* were the newspapers that reported on health care apps most frequently; in the United States, health care apps were most frequently reported in the *New York Times* and the *Washington Post*.

**Figure 3:**
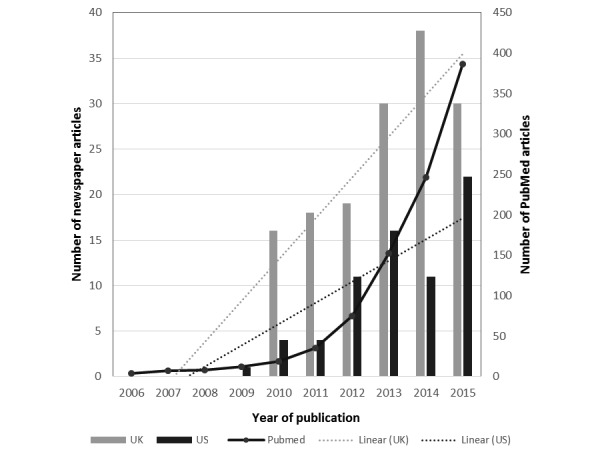
Number of articles about health-related apps in UK and US newspapers (left axis), and in PubMed (right axis) from 2005 to 2016.

In the United Kingdom, the number of published articles on health care apps increased notably over the study period, particularly during 2014, whereas in the US newspapers, the articles reached peak incidence in 2013 and 2015. The overall trends for both UK and US newspaper articles were upward across the study period as shown in Figure 3. Based on study title and abstract, a total of 944 research articles that involved health care apps were retrieved from PubMed over the study period. As indicated in Figure 3, the number of the scientific articles increased exponentially over the study period.

### Newspaper Section

Of the 220 identified newspaper articles, 58 articles (26.4%) appeared in the features section and 42 articles (19.1%) appeared in the main news section. Less than 10% of the articles (19 articles) appeared in the health/life sections of the newspapers included in the evaluation. Although less frequently, health care apps also appeared in the business/financial section (13.2%, 29/220) and editorial section (1.8%, 4/220) of the newspapers.

### Interrater Agreement

The calculated interrater reliability (Cohen kappa) agreement between the codes from the two coders were all positive and ranged from .421 to .889. Cohen suggested the kappa result be interpreted as follows: values ≤0 as indicating no agreement, .01-.20 as none to slight, .21-.40 as fair, .41-.60 as moderate, .61-.80 as substantial, and .81-1.00 as almost perfect agreement [38]. Accordingly, the current interrater analysis revealed that seven of 19 items (37%) illustrated moderate agreement, seven items (37%) illustrated substantial agreement, and five items (26%) illustrated almost perfect agreement between the two coders, demonstrating objectivity in elemental judgment as illustrated in Table 1.

### Content of Newspaper Article

Within the 220 articles selected, the most frequent areas covered in the newspapers were general information about health care apps (101 articles; 45.9%), sport and fitness apps (63 articles; 28.6%), and disease-specific apps (20 articles; 9.1%). The remaining articles (36 articles; 16.4%) covered a range of topics, such as apps as sources of information, diet/healthy food apps, and apps used to communicate between health care professionals and between patients and health care professionals (Table 2). Most articles (Table 3) were written from a societal perspective (159 articles; 72.3%) followed by an industry point of view (30 articles; 13.6%); the other viewpoints included policy-related (16 articles; 7.3%), scientific (8 articles; 3.6%), legal (4 articles; 1.8%), and economic perspectives (2 articles; 0.9%). There was no significant difference between the key perspective of UK and US articles (χ^2^_6_=6.5, *P*=.36).

**Table 1:** Kappa values and interrater agreement for the coding of newspaper articles (n=10).

Item	Kappa value	Agreement (%)
Health care apps is main theme	.45	91
Other health care-related themes covered	.90	96
Key perspective	.46	91
First mention of health care app	.58	86
Linked disease (or group of diseases)	.80	82
Main voice of information	.50	68
Type of benefits of health care-related use of the mobile app(s)	.81	95
Type of harm or risk of health care-related use of the mobile apps	.53	91
Are direct quotes about health care app(s) used in the article?	.62	82
Barrier to the use of the health care app in routine clinical practice stated	.81	91
Facilitators to the use of the health care app in routine clinical practice stated	.75	86
Who or what is cited as the main source of information for the newspaper article?	.61	68
Article slant about health care-related app in this article	.63	82
Is the main claim about the health care app(s) in newspaper article/presentation style?	.49	86
The quality of information presented from the researcher’s perspective	.69	95
Main theme regarding the use of health care app(s) in this article	.88	91
Relevant apps mentioned in the newspaper can be downloaded on operating mobile system	.42	82
Benefit or advantage of health care-related use of the mobile app(s)	.62	95
Potential harm or risk of health care-related use of the mobile app(s)	.63	91

**Table 2:** The main themes of the reviewed newspaper articles (N=220).

Main theme	United Kingdom articles (n=151), n (%)	United States articles (n=69), n (%)	Total (N=220), n (%)
Other	1 (0.7)	3 (4.3)	4 (1.8)
Communication tool	1 (0.7)	2 (2.9)	3 (1.4)
Diet/healthy food	11 (7.3)	5 (7.2)	16 (7.3)
Disease specific	16 (10.6)	4 (5.8)	20 (9.1)
Information source	9 (6.0)	4 (5.8)	13 (5.9)
Sport and fitness	43 (28.5)	20 (29.0)	63 (28.6)
Health care apps	70 (46.4)	31 (44.9)	101 (45.9)

**Table 3:** The key perspectives of the reviewed newspaper articles.

Key perspective	United Kingdom articles (n=151), n (%)	United States articles (n=69), n (%)	Total (N=220), n (%)
Political	0 (0.0)	0 (0.0)	0 (0.0)
Other	1 (0.7)	0 (0.0)	1 (0.5)
Economic	1 (0.7)	1 (1.4)	2 (0.9)
Legal	3 (2.0)	1 (1.4)	4 (1.8)
Scientific	4 (2.6)	4 (5.8)	8 (3.6)
Policy related	6 (4.0)	10 (14.5)	16 (7.3)
Industry	22 (14.6)	8 (11.6)	30 (13.6)
Societal	114 (75.5)	45 (65.2)	159 (72.3)

**Table 4:** The frequency of mentioning health conditions related to health care apps in newspaper articles.

Medical condition (*British National Formulary*, 65th edition, 2014, classification)	Frequency, n (%)	
	United Kingdom (n=75)	United States (n=44)
Cardiovascular	17 (22.7)	11 (25.0)
Central nervous system	8 (10.7)	3 (6.8)
Respiratory system	4 (5.3)	2 (4.5)
Gastrointestinal system	0 (0.0)	1 (2.3)
Endocrine	19 (25.3)	12 (27.3)
Cancer	9 (12.0)	1 (2.3)
Infection	3 (4.0)	1 (2.3)
Eye	4 (5.3)	1 (2.3)
Skin	5 (6.7)	3 (6.8)
Ears, nose, and throat	4 (5.3)	4 (9.1)
Obstetrics and gynecology	2 (2.7)	2 (4.5)
Other	3 (4.0)	3 (6.8)

Although most of the articles (124 articles; 56.4%) did not refer to a specific mobile operating system, 58 articles (26.4%) mentioned iOS (Apple) as the operating system; both Apple and Android operating systems were mentioned together in 42 articles (19.1%). There was no significant difference in the reporting of mobile operating systems between the United Kingdom and United States (Mann-Whitney *U*=12.0, *P*=.39).

Of the 220 articles, approximately 106 articles (48.2%) related to general health. including smoking cessation, alcohol use, weight loss, fitness. and exercise tracking, whereas 81 (36.8%) were linked to specific diseases. A smaller number of articles (33 articles; 15.0%) mentioned multiple diseases and/or medical uses (eg, first aid apps, thermometer apps, ear examination apps, diabetes apps).

Endocrine diseases (mainly diabetes) were frequently mentioned in relation to the use of health care apps; however, cardiovascular diseases (including blood pressure and heart rate monitoring) were the most commonly mentioned conditions in the relevant newspaper articles (Table 4). There was no difference between UK and US newspapers in terms of the frequency of mentioning specific disease indications (Mann-Whitney *U*=41.5; *P*=.08).

The vast majority of the newspaper articles (201 articles; 91.4%) mentioned at least one benefit related to the use of health care apps, whereas only 53 articles (24.1%) reported at least one risk. There was no significant difference in the frequency of benefits or risks reported between the UK and US articles (χ^2^_1_=1.3, *P*=.44). The three main benefits associated with the use of health care apps related to personalized care were improvement in general health and fitness (in 124 articles; 56.3%), public/patient access to health information (in 32 articles; 14.5%), and improved health outcomes for the public/patients (in 32 articles; 14.5%). Conversely, a breach in confidentiality was the most commonly cited risk (in 25 articles; 11.3%). In addition, approximately 9% of the articles (20 articles) reported that health anxiety could be induced because of the use of health care apps (Table 5). For example, in one of *The Guardian* articles, it was debated whether fitness tracking apps are “untested and unscientific and they open a door of uncertainty,” and uncertainty may ignite anxiety in people [39].

Approximately half of the articles (101 articles; 45.9%) mentioned potential facilitators to the use of health care apps, whereas less than 10% of articles (16 articles) reported real or potential barriers to their use. The main recorded facilitator was improved technology (87 articles; 39.5%). No difference was found between UK and US newspapers in terms of the frequency of reporting of facilitators or barriers to the use of health care apps (*P*=.12) as shown in Table 6.

Most of the articles had a positive slant regarding the use of health care apps (146 articles; 66.4%), whereas 26.4% (58 articles) were classified as having a mixed slant. Relatively few articles reported negative views (11 articles; 5.0%) or a neutral view (5 articles; 2.2%). The majority of the articles (184 articles; 83.6%) were judged as having balanced judgments (ie, the authors did not exaggerate or understate the main message). The quality of the information presented in the newspaper articles was classified into three categories: poor, average, and excellent. Articles were classified as poor if judgment was not balanced and/or if only anecdotal evidence was presented; articles were classified as excellent if they presented information based on firm evidence, often also presenting quotations from experts in the field. Overall, 132 articles (60.0%) were scored as having poor information about health care apps, 75 articles (34.1%) were judged to have good quality information, and 13 articles (5.9%) were judged to provide excellent quality information. There was no significant difference between the quality of the reporting process in UK articles and US articles (χ^2^_2_=1.8, *P*=.29).

**Table 5:** Summary of the mentioned benefits and risks associated with using health care apps in newspaper articles.

Benefits and risks mentioned in newspaper articles		Frequency, n (%)	
		United Kingdom	United States
**Benefits (n=222)**		n=151	n=71
	Personalized care	88 (58.3)	36 (50.7)
	Public/patient access to health information	25 (16.6)	7 (9.9)
	Improved health outcomes for public/patients	20 (13.2)	12 (16.9)
	Communication between public/patients and health care providers	7 (4.6)	7 (9.9)
	Public/patient satisfaction	6 (4.0)	7 (9.9)
	Economic benefit to society	2 (1.3)	1 (1.4)
	Communication among public/patients	1 (0.7)	1 (1.4)
	Communication among health care providers	0 (0.0)	0 (0.0)
	Other	2 (1.3)	0 (0.0)
**Risks (n=60)**		n=36	n=24
	Anxiety	15 (41.7)	5 (20.8)
	Confidentiality	16 (44.4)	9 (37.5)
	Deterioration of outcome	2 (5.6)	10 (41.7)
	Other	3 (8.3)	0 (0.0)

**Table 6:** Summary of the facilitators and barriers mentioned in the newspaper articles.

Facilitators and barriers mentioned in the newspaper articles		Frequency, n (%)	
		United Kingdom	United States
**Facilitators (n=101)**		n=61	n=40
	Access	1 (1.6)	2 (5.0)
	Commercial	2 (3.3)	0 (0.0)
	Evidence to support adoption	2 (3.3)	1 (2.5)
	Positive beliefs	2 (3.3)	2 (5.0)
	Technology	52 (85.2)	35 (87.5)
	Other	2 (3.3)	0 (0.0)
**Barriers (n=16)**		n=10	n=6
	Access	2 (20.0)	0 (0.0)
	Commercial	1 (10.0)	3 (50.0)
	Lack of evidence	2 (20.0)	1 (16.7)
	Negative beliefs	4 (40.0)	1 (17.7)
	Other	1 (10.0)	1 (17.7)

## Discussion

### Methodology Used

Newspapers are read by a high proportion of the population in the United Kingdom and United States on a regular basis. There is also a strong correlation between newspaper publishing and other mass media platform reporting on the same topic or issue [40]. The methodology adopted for this research was based on earlier published research using content analysis of newspapers [41-44]. In this study, there was a substantial level of agreement between coders as the median kappa statistic score among the two researchers for all variables was .624 (range .421-.889). This indicated moderate to almost perfect agreement for all coding variables [35]. This finding is similar to the level of interrater agreement previously reported in published research on content analysis of newspaper articles [45,46]. The validity of this study was enhanced by precoding 10 articles followed by adjustment to the coding framework. There is no specific requirement for a particular sample size in kappa calculations. In this study, a random sample of 10% of the published articles was included.

### Frequency of Relevant Articles

A total of 220 articles were identified in the highest circulation of UK and US newspapers over the period of 10 years investigated in the study (2006-2015). For the same period, the number of scientific articles published in PubMed was 944, with the number increasing exponentially over time. In September 2013, the FDA issued guidance relating to medical apps that stated that the majority of health care apps pose a low risk for users or patients and indicated that they would only get involved in regulating those mobile apps that transform mobile devices into medical devices [47,48]. This may have caused the decline in the number of articles published about health care apps in 2014 in the United States. For the United Kingdom, establishing the NHS health app library in 2013 led to a more widespread consideration of health apps; this was reflected the relatively high reporting in UK newspapers in that year (Figure 2).

### Content of the Selected Articles

The main theme of articles on health care apps was found to be related to public health and well-being, including lifestyle management. Unsurprisingly, the related matters of sport and fitness were frequently reported themes. Many of the articles were reported from a societal perspective. This was expected because a high proportion of the newspaper articles focused on general public health topics, including fitness, well-being, and good diet. This finding concords with the finding of a recent report which stated that apps that remind, monitor, and track are intended for the general public, as is the case with social media apps [49]. These kinds of innovative apps can play an important role in general health and lifestyle and can help motivate people to make healthy lifestyle choices [3]. In 2014, a study was conducted to identify the number of health and fitness apps in the Apple Store and Google Play. The author found 23,490 and 17,756 health and fitness apps available in the Apple Store and Google Play, respectively. Moreover, the number of health and fitness apps had grown by 62% over the previous year (2014) in comparison with a 33% growth for apps in general [50]. This vast number of available health and fitness apps helps explain why such apps were reported more frequently in the media when compared with disease-specific apps.

Diabetes was the illness for which apps were most frequently reported by the newspapers; other diseases frequently mentioned were cardiovascular disease and those affecting the central nervous system. A total of 422 million people have been diagnosed with diabetes worldwide and the number is increasing in many countries due to an increasing age demographic together with an increased incidence of obesity and low physical activity [51]. Diabetes is a progressive disease that is associated with several complications including cardiovascular, kidney, and eye complications. Control of diabetes requires self-monitoring of blood glucose levels. Advanced mobile technology allows patients to record these blood levels electronically. Some available apps provide several advantages to manual recording including graphing daily blood sugar levels together with calorie intake and exercise undertaken [52]. A recent review of diabetes-related apps recorded a range of functions that these apps can perform, including documentation, data forwarding, information provision, analysis, recipe suggestions, reminder, and advisory functions [53]. Despite the high prevalence of asthma worldwide, reporting on apps for respiratory illness was infrequent. Available apps in this domain are used to record symptoms of asthma, peak flow readings, and number of attacks, and to provide information about asthma triggers.

As anticipated, in this study the benefits of health care apps were more frequently discussed within articles than were risks. Improved personalized health and fitness were the main benefits described and the issue of confidentiality was the most frequent risk discussed. The literature has documented that newspapers in general overreport the benefits and underestimate the risks when publishing information about health intervention research [47,54]. If the benefits of health care apps are overstated, this could raise public expectations about improved health, whereas overstating risk could generate anxiety and may negatively affect the uptake of what could be a helpful intervention.

In this study, the quality of reporting on health care apps was judged to be poor in most of the articles (60%). These articles in general were brief and did not add sufficient supporting evidence or refer to health agencies. A third of the articles were classified as having average/good quality of reporting, whereas only 5.9% were considered excellent. This was consistent with previous research articles that highlighted the general poor quality of information in the print media about health-related matters [55,56]. It has been reported that high quality newspaper articles often flow from a press release, generally from a scientific journal [19]. Therefore, researchers in the field should ensure that such press releases are written alongside their publications in order to help better information reach the public via the mass media.

There are several limitations to this study. This study examined reporting on health care apps in newspapers only and did not cover other mass media sources such as television and radio. However, as mentioned earlier, it has been reported that the content of newspaper articles correlates closely with other media sources [57]. In addition, some highly circulated US newspapers such as the *Dallas Morning News*, *Chicago Tribune*, and *Chicago Sun-Times* were not included in the study because they were not archived by Nexis. Moreover, limited articles were accessible in Nexis for the *Los Angeles Times* and *Newsday* as only articles from the previous 6-month period were available.

### Conclusions

This study has revealed that health care apps are indeed reported on by UK and US newspapers. The number of articles reporting on health care-related apps increased over time in UK and US newspapers over the 10-year study period. Several benefits were reported relating to app use, especially in relation to promotion of a healthy lifestyle, whereas reporting on risk was less frequent. Improving personalized care was the most frequently mentioned benefit; confidentiality breaches were the most commonly reported risk. Diabetes was the disease most commonly linked with the use of health care apps. In general, the main claims about benefits had a positive slant, but articles generally were balanced in their judgment.

## Abbreviations

FDA: Food and Drug Administration
NHS: National Health Service
